# The role of patient care workers in private hospitals in the Cape Metropole, South Africa

**DOI:** 10.4102/curationis.v40i1.1704

**Published:** 2017-07-31

**Authors:** Louise A. Aylward, Talitha Crowley, Ethelwynn L. Stellenberg

**Affiliations:** 1 Department of Nursing and Midwifery, Faculty of Medicine and Health Sciences, Stellenbosch University, South Africa

## Abstract

**Background:**

Nursing managers have to meet expectations of patients despite economic pressures, an increasing burden of disease and nursing shortages. Shifting health care-related tasks to lower categories of staff, including non-nursing support staff, has become one solution to address this dilemma. Patient care workers are a specific group of non-nursing support staff working in South African hospitals. Although patient care workers have been used for several years and their numbers are increasing, there are controversial opinions about the role of patient care workers, ranging from praise for their contribution towards patient care to serious concerns about the impact of their role on patient safety.

**Objective:**

The study objective was to explore and describe the role of patient care workers in private hospitals.

**Methods:**

A qualitative, descriptive design was applied to explore the role of patient care workers. Purposive sampling was used to select unit managers, nurses and patient care workers from medical and surgical wards of three private hospitals. Fifteen semi-structured interviews were conducted and transcribed verbatim. The researcher applied interpretative data analysis to move from the participants’ descriptions of their experiences to a synthesis of all participants’ descriptions.

**Results:**

Patient care workers are involved in direct patient care and spend much time with patients, often not working under direct supervision of registered nurses despite limited training and lack of regulation. Their contribution, however, is valued by nurses.

**Conclusion:**

Patient care workers are well-integrated into the patient care team and are mostly seen as nurses. Yet, there are concerns about their evolving role despite their limited training and the lack of direct supervision. Regulating the work of patient care workers is recommended.

## Introduction and background

The realities of the 21st century affect the expectations from nurses, for example, that nurses have to do more with less and have to ensure a good overall experience by the patient (Armstrong et al. 2013). Economic pressure, an increasing burden of disease and the continuing nursing shortage are some of these realities. Task shifting has been identified as one of the solutions to meet expectations despite these challenges (Armstrong et al. 2013).

Task shifting in nursing refers to the delegation of health care activities from higher to lower categories or to non-nursing support staff. The aim is to ease the workload of each nurse involved while simultaneously ensuring quality patient care (Callaghan, Ford & Schneider 2010).

The use of non-nursing support staff can be traced back to the beginning of modern nursing, from the presence of nurses’ aides during the Crimean War from 1854 to 1856, through to the auxiliary nurse role of the mid-1950s and to the health care assistant (HCA) role which was identified around 1990 in the United Kingdom (UK) (Hand 2012).

Although HCAs in the UK have variable job descriptions, depending on the organisation providing employment, their work is usually focused on patient care. The period of their training varies from two weeks to two years (Furåker 2008). They are not regulated, but the UK Coalition Government is proposing a system of voluntary regulation for health care support workers and is in the process of developing codes and standards as a step towards regulation (Hand 2012).

In Scotland and Wales, there are codes of conduct for HCAs and a code of practice for employers. Scotland also has induction standards, while Wales gives guidance regarding induction best practices. These employer-led methods of regulation were brought into practice in 2011 (Hand 2012).

In Sweden, the role of HCAs has been regulated through legislation since the 1950s. Their work is oriented to support the registered nurse and is focused on direct patient care and housekeeping duties (Furåker 2008). They have a three-year upper secondary school education in health care (Furåker 2008).

In New Zealand, a decision document states that the HCA’s role incorporates both patient support and tasks related to maintaining the physical environment. However, concerns were raised regarding the balance between direct care and housekeeping roles, the insufficiency of the training course and fear that the HCA’s role would encroach on the enrolled nurse’s (EN’s) scope of practice (Acute care HCA duties at Wairu Hospital 2012). International literature, therefore, confirms that the HCA’s training, role and regulation are neither clarified nor standardised.

In South Africa, unlike most other countries, auxiliary nurses are regulated by the South African Nursing Council (SANC). According to the *Nursing Act*, No. 33 of 2005 (South African Nursing Council 2005), auxiliary nurses are persons educated to provide elementary nursing care as directed and supervised by a professional nurse (PN) and the former, therefore, assist PNs to provide patient care. Elementary nursing includes ‘practical self-care and activities of daily living interventions that assist the health care users to promote and maintain their health status through the application of prescribed standards of care’ (South African Nursing Council 2013).

In recent years, hospitals have been employing patient care workers (PCWs) to assist nurses. They are not regulated and have no formal nursing training (Stellenberg & Dorse 2014). Several training institutions in South Africa provide training, ranging from three to six months to equip care workers to provide home based care, although they are also employed in hospitals. The training programme typically includes basic knowledge of the most common types of debilitating and terminal diseases, the normal process of ageing and being able to recognise when referral is needed (South African Quality Assurance 2012).

The researcher observed that there is a tendency to increase the number of PCWs in private hospitals, although there are critical voices raised against their employment and the possible risks to patient safety. Dorse (2008) mentions that their increased use is not based on best practice, but a strategy to address the shortage of qualified staff and of health care inflation.

### Problem statement

Patient care workers have been employed in private hospitals in South Africa for several years, but their role remains controversial and not clearly described. The researcher observed that this uncertainty about their role leads to either resistance to their employment or to potentially ineffective or unsafe use of the PCWs already employed in hospitals. It may further be a potential risk for patient care and a challenge to nursing management. There is a lack of evidence about the role of PCWs in the South African setting.

Biddle (1986) describes ‘role’ as the social position of a person and the rights, obligations and expected behaviour of this person and of other persons in the particular social setting. In this study, the ‘role’ of PCWs refers to their position in the patient care team, their supervision and activities.

### Aim

The aim of this study was therefore to explore and describe the role of PCWs in private hospitals in the Cape Metropole, South Africa. The specific objectives included exploring their activities, supervision, position in the patient care team, reasons for PCW employment and concerns about the role of PCWs.

## Research design

An explorative descriptive qualitative design was used. This design enabled the researcher to explore the role of PCWs as it naturally occurs and describes how participants ascribed meaning to their interactions with PCWs.

### Setting

The research was conducted in medical and surgical wards in three private hospitals in the Cape Metropole in the Western Cape, South Africa. The researcher observed that most PCWs are utilised in the medical and surgical wards of hospitals, thus the focus on these wards in this study.

### Population and sample

Employees who were permanently employed in medical or surgical wards and who had more than one year experience of working with or as a PCW were included in the study. This included a total of 18 unit managers (UMs), 54 PNs, 90 nurses (enrolled and auxiliary) and 36 PCWs. Purposive sampling was applied to select participants for individual interviews (*n* = 15), involving one UM, one PN, one other nurse (either an EN or enrolled nurse auxiliary, ENA) and two PCWs from each hospital. This was done to include the three hospitals and the perspectives of all categories. Data saturation was achieved after 12 interviews, but the researcher continued to interview all of the 15 participants in order to include all categories and hospitals.

### Data collection

A semi-structured interview guide with open questions and additional probing words, based on the literature and focused on the objectives of the study, was used. Fifteen interviews were conducted by the first and second author. An open-ended question, ‘What is the role of PCWs in your ward?’ was asked, with further probing questions, for example ‘What do PCWs do from the time that they come on duty until the end of the shift?’ In addition, demographic data of participants were collected prior to each interview.

The pilot interview involved one ENA from a target hospital. Data from the pilot interview was not included in the final analysis of the study. No changes were made to the interview guide, although probing questions were added as the study progressed.

Interviews were conducted and audio recorded in private and comfortable venues in each of the three hospitals, at dates and times suitable to the participants. Each interview started with a short summary of what the interview and study was about. The rights of the participant were explained, specifically regarding the confidentiality of the information and the right to freely participate and to withdraw from the study at any point.

### Data analysis

Audio recordings were transcribed verbatim. Each sentence or meaningful section of the recording was captured in a separate row. Unique identifiers and columns for notes were used to aid analysis of data. Interpretative data analysis, as described by Terre Blanche, Durrheim and Painter (2006), was applied to find commonalities and differences between interviews and then to group these broader categories in sub-themes and themes that represented most of the data. The five data analysis steps, namely familiarisation and immersion, inducing themes, coding, elaboration and interpretation, and checking were used to uncover the role of PCWs. The second author assisted with interviews and evaluated consistency of data coding. Audio recordings and transcriptions of data and the documentation of the research process were carefully kept should the research process or outputs need to be examined.

### Ethical consideration

To ensure an ethical foundation, permission to conduct this study was obtained from the Health Research Ethics Committee of the University of Stellenbosch (Ethics Reference: S14/02/04) and the research boards of the three hospital groups. Informed written consent was obtained from all the participants. Access to data was restricted to the authors and the data transcriber, who signed a non-disclosure agreement. Data were password protected and stored securely. Confidentiality of participants was ensured by using pseudonyms and by not connecting responses to individuals or hospitals.

## Results

Of the 15 participants, 9 were employed in medical wards and 6 in surgical wards. The average duration of employment as a nurse or PCW was 12 years, while the average duration of working with or as PCWs was 8 years. The average age of participants was 40 years. Fourteen participants were female. Five key themes and several sub-themes emerged, as depicted in Box 1.

BOX 1 Themes and sub-themes.

**Table:** 

Themes	Sub-themes
Activities of PCWs	Indirect patient care Direct patient care
Organisation of PCWs’ tasks	Supervision of PCWs Allocation of tasks Reporting structure
PCWs’ position in the patient care team	Value of PCWs Seen as nurses versus not seen as nurses Compared to ENAs
Reasons for employment of PCWs	Preventing patient falls Assisting nurses Preventing family complaints Institutional financial constraints
Concerns about the PCW role	Limited appreciation for PCWs Lack of career development opportunities for PCWs Variety in PCW training courses PCWs are doing more than they are allowed to PCWs may lack insight

*Source:* Authors’ own work

PCW, patient care workers; ENA, enrolled nurse auxiliary.

### Activities of patient care workers

Two sub-themes emerged, namely indirect patient care and direct patient care.

Although PCWs spent most of their time on direct patient care, they were sometimes used for indirect patient care activities including secretarial work when the ward secretary was absent, controlling stock, housekeeping, portering and errands.

‘It’s a care worker that is appointed to help with stock. Sometimes if he has finished the stock taking, he helps in the ward.’ (Participant 11, female, EN)

‘We (PCWs) have to clean around the patients … see that the family member doesn’t come in a dirty place.’ (Participant 2, female, PCW)

Direct patient care focused on keeping patients clean, comfortable and safe. This allowed them to observe and to report abnormalities and changes, including interacting with patients and families. Although these activities were described as basic care, participants mentioned the intimate nature thereof. Most participants expressed that these were nursing activities and that the PCWs were actually nursing and nurturing the patients.

‘The care worker actually sees that the patient is clean; that their surroundings are clean and yes, make the patient comfortable. That’s actually the care worker’s role.’ (Participant 4, female, PCW)

‘And you know who they [*the patients*] compliment? The carers, when they go home, because they are the people who interact with them. Because we [*PNs*] don’t really interact with the patients.’ (Participant 12, female, PN)

The discipline of record-keeping of activities was described by a PCW:

‘Whatever you do you write down; even if you give a glass of water you write down.’ (Participant 7, female, PCW)

Patient care workers were sometimes pressurised to do more, for example to monitor vital signs and to do diagnostic tests, for example urine analysis. All UMs were concerned that PCWs might do more than they should do. All PNs reported that PCWs actually did more than they should have done. Yet, PNs did not express concern about this, as illustrated by a PN:

‘You will use, if you have, an extra care worker to go and do the observations, but they do help us; they can do it, because everything is machine wise.’ (Participant 1, female, PN)

### Organisation of patient care worker tasks

The following sub-themes were induced regarding the organisation of PCW tasks: supervision of PCWs, allocation of tasks and their reporting structure.

It was mentioned of PCWs that ‘they are not allowed to basically be alone with a patient’ (Participant 3, female, UM) and that they were supposed to do basic care under supervision of a PN. However, participants also mentioned that PCWs often worked on their own and would ask for help from nurses or another PCW when needed. Although nurses worked with the PCWs in the ward, they were not present with the PCW during basic care activities, as stated by a UM:

‘Now, in essence, they are under supervision because there is a sister or two sisters and me. … But they are not there when they take patients out of the bed and put them into the chair.’ (Participant 8, female, UM)

All participants reported that PCWs were included in meetings for shift handover during which tasks were delegated to them. The PCW would be allocated to a group of patients or to a specific patient to supervise that patient. Patient and task allocation were indicated in an allocation book. Yet, ‘most of the time they know what to do and they follow a routine’ (Participant 11, female, EN). Routines included bathing and feeding patients, and tidying the environment.

Patient care workers often referred to their responsibility of reporting abnormalities or concerns to nurses, such as pain and general deterioration of a patient’s condition. Reporting to the PN was often mentioned, as well as reporting to the nearest nurse or the nurse responsible for either the patient or for medication administration. Nurses depended on this reporting and mentioned that PCWs were good at reporting.

‘I firstly report to that person who is responsible for that patient. If that person is not there then I go to a senior sister or the unit manager.’ (Participant 4, female, PCW)

‘And most important they know whatever observation, whatever things they do to the patients, whatever procedures, they must report.’ (Participant 11, female, EN)

However, some nurses raised a concern that PCWs might not have enough knowledge to identify abnormalities. One PN mentioned that she (the PN) insisted to be called to evaluate an abnormality, such as skin lesions.

‘Every time she [*the PCW*] works with the patient she has to call me, “Sister, come and see for yourself”.’ (Participant 10, female, PN)

### Patient care workers’ position in the patient care team

This theme relates to how PCWs fit into the patient care team, and includes the value of PCWs, how they are seen as nurses versus not seen as nurses and how they are compared to ENAs.

All participants agreed that PCWs added value in the ward. Several terms to express this were used, including: ‘The care workers are really important’ and they are ‘a very, very big help’ (Participant 12, female, PN).

Most participating nurses and PCWs were clear that they viewed PCWs as nurses and what they do as nursing care. A PN explained:

‘You will call them nurses all over because they are such a part of the whole team and nursing, because how can you care for a patient and not do nursing.’ (Participant 1, female, PN)

They did not observe much difference between the roles of the PCW and the ENA.

‘And the care workers in this ward are almost like the ENAs.’ (Participant 1, female, PN)

‘…Because there are some individuals who are equal to ENAs.’ (Participant 12, female, PN)

However, one UM was clear that PCWs were not nurses and expressed her concern that nurses saw PCWs as nurses and consequently allocated nursing tasks to them.

‘What I also pick up which is a concern to me specially in the medical ward, is that nurses do tend to think that the care workers are also nurses, so they almost expect the care worker to know exactly and to do what they are doing.’ (Participant 3, female, UM)

PCWs further reported that patients addressed them as if they were nurses. A PCW elaborated:

‘And the patients … because they don’t know which category I am, they just call you a nurse.’ (Participant 13, female, PCW)

However, a UM explained her discomfort when having to rectify a patient’s misperception:

‘Implications for me would be a patient being unhappy with something that’s being done and me having to tell the patient that that person is actually not a nurse.’ (Participant 3, female, UM)

### Reasons for employment of patient care workers

Participants discussed several reasons for the employment of PCWs, including the prevention of patient falls, assistance to nurses handling a heavy workload, prevention of complaints and institutional financial constraints.

Initially, PCWs were used primarily to keep the confused and elderly patients safe. This was mentioned as a major task allocated to PCWs. Additional PCWs were being hired through agencies when there were confused patients in the ward. The importance and outcome of this intervention were noted by a PN:

‘You know why the carers are also important lately? Because there are less falls now also.’ (Participant 12, female, PN)

All PCWs further described the reason for their employment as relating to ‘do those types of things that nurses won’t be able to get to’ (Participant 2, female, PCW). ENAs were described as being busy with monitoring vital signs and admissions, while ENs were occupied with the administration of medication and PNs with doctors’ rounds, while PCWs ‘know everything the patient needs’ (Participant 7, female, PCW).

‘We are with the patient 12 hours. Sometimes the sister doesn’t get time to get to the patient during the day; she is so busy, but we are there and we keep them comfortable.…’ (Participant 9, female, PCW)

A PN explained the reason for employment of PCWs as ‘the complaints also that we get from the family’ (Participant 12, female, PN). A UM further described how PCWs were responsible to do hourly nappy checks and to record their findings per hour, as family members complained that their parents were not being kept clean and dry. The UM could use the record as evidence when complaints were lodged.

Three participants referred to financial reasons for the employment of PCWs. An EN’s opinion was: ‘I think it’s financial, otherwise they would just employ ENAs’ (Participant 5, male, EN). A UM further referred to budgetary constraints being considered when deciding to hire PCWs, but that she would not use a PCW when ‘a skilled hand is needed’ (Participant 15, female, UM). PCWs earned less than ENAs, yet they almost did the same work.

Participants further were of the opinion that the reasons for employment of PCWs had changed over time. A UM explained: ‘initially they were just there to assist us with the confused patients’ (Participant 3, female, UM). However, PCWs were also employed to assist nurses with nursing activities and for financial reasons.

### Concerns about the patient care worker role

The concerns expressed by participants included limited appreciation for PCWs, a lack of career development opportunities for PCWs, the variety in PCW training courses, that PCWs are doing more than they are allowed to and that PCWs may lack insight.

Participants mentioned that PCWs were not appreciated enough and were seen as cheap labour.

‘They must definitely be paid more.’ (Participant 12, female, PN)

It was said that although their colleagues gave recognition to PCWs, the same was not true of management, demonstrated specifically by their exclusion from International Nurses’ Day activities.

‘Every year the 11th or 12th of May we have a National Nursing Day and they [*PCWs*] are not invited, because they are not nurses and with that we have got a problem. So what we do we go on the day and we go and if we have sweeties or so, your whole pocket is full of sweeties. You bring it back because you feel terrible because they are nurses; in our eyes they’re nurses.’ (Participant 1, female, PN)

Patient care workers further mentioned that they could not enhance their career if they did not have a Senior Certificate. Yet, a few participants mentioned, with much appreciation, that they were working with nursing students or qualified nurses who started their career as PCWs. However, a UM voiced her concern that PCWs working in the hospital have the expectancy to do a nursing course although they might not have the ability to do what was expected of ENAs.

‘Yesterday we were introduced to the new students, who start their bridging course for registered nurse. And [name] was one of the first carers we employed and she is now doing the bridging course. And I started crying.’ (Participant 8, female, UM)

‘Some of them [*PCWs*] might have the expectation to go on, but then you can see when they are working in the ward, that they won’t have the ability. They must be realistic about that.’ (Participant 15, female, UM)

A UM further explained her concern about the variety of courses completed by the PCWs who applied for positions in her ward and that ‘the training is not the same’ (Participant 3, female, UM). She, therefore, suggested a bridging course for PCWs, including training on how to work in a private setting and the ethics involved in the caring of patients. Some participants proposed regulation, ‘to know what is expected from the care worker’ (Participant 3, female, UM).

Although there seemed to be a fair amount of clarity about the PCW’s role, all UMs and PNs indicated that PCWs were doing more than they were allowed to do. A UM explained that PCWs were initially employed to do basic activities, for example giving water to a patient and answering patient calls. However, they are now expected to be scientific in their approach.

‘And we expect them to be scientific, look at a patient’s sacral area and say it is red. We do expect them to document that it is red or the skin is broken and where exactly, where it is and it is one centimetre.’ (Participant 8, female, UM)

Another UM indicated that nurses see the PCWs as nurses and, therefore, expect nursing tasks from them. However, although UMs seemed concerned about this, PNs apparently accepted that the role of the PCW was expanding. A PN mentioned:

‘Then we tell them, in this ward, if we are really short they help us with everything.’ (Participant 1, female, PN)

Concern was expressed that PCWs might be able to do the tasks but lack insight to understand the possible consequences of not doing what is expected of them. Three reasons for the expanding role of PCWs emerged. Firstly, PCWs were pressurised by nurses to take on more tasks, either because the wards were busy and nurses could not get around to do everything they had to do, or because nurses expected that PCWs should be able to do more, for example monitor vital signs.

‘The other thing is you, you can easily be pressurised, that when the ward is getting busy, you can easily be pressurised into allocating a different task to that care worker. So you can easily trust her and say okay, carry on with the observations, and that is where the problem lies.’ (Participant 15, female, UM)

Secondly, PCWs learned how to do things and used the opportunity to use their new skills. Thirdly, being allowed to take on more tasks was seen as recognition that the PCWs were not ‘ignorant’ and that they were indeed nurses.

## Trustworthiness

The accuracy and truthfulness of the findings were ensured through the application of the principles of credibility, transferability, dependability and conformability (De Vos et al. 2011).

Credibility in this study was established through the checking of transcriptions of audio recordings for accuracy by the first and second authors. Rich data, including selected quotes from the interviews, were used in the report for validation by the reader. The decision to transfer findings to other settings is supported by a description of the setting and the research process that was followed. In addition, multiple perspectives were used to illuminate the role of PCWs, including the perspectives of UMs, nurses and PCWs from three different private hospital groups.

Dependability was enhanced by providing a detailed description of the research design, process and interview questions to enable future researchers to follow a similar research approach. To support confirmability, the authors ensured that documentation and audio recordings of data and the research process were carefully kept to provide an audit trail. This enables examination of both the research process and research outputs.

## Discussion

All participants were clear that PCWs were primarily tasked to provide direct care to patients, therefore spending most of their day close to patients. Most participants also mentioned that PCWs did more than they were allowed to do, which was a concern for UMs. Their tasks involved all aspects of hygiene and comfort, similar to the activities of daily living or physiological aspects of care, referred to by both Henderson and Orem in George (2010) in their theories of nursing. This supports the notion by McKenna, Hasson and Keeney (2004) that many of the core skills of nursing have been handed over to PCWs. Even though UMs made an initial effort to explain that PCWs did not do nursing by labelling their work as ‘non-nursing tasks’ and ‘non-scientific nursing’, most participants eventually concluded that PCWs were seen as nurses and that they were actually performing basic nursing tasks. Their activities, therefore, overlap with those of ENAs, although their training is not standardised and they are not regulated in terms of the *Nursing Act*, No. 33 of 2005. According to Stellenberg and Dorse (2014), the practice of PCWs in hospitals may put patient safety at risk and may lead to PNs being confronted by ethical and legal issues.

Participants also explained the intimate nature of PCWs’ activities and that they spent much time close to patients. Their closeness to patients allowed PCWs to observe the patient’s condition. It was evident that the expectancy was that they would report abnormalities observed to nurses. Most nurses mentioned that PCWs were good observers, although concern about their lack of insight in their actions and observations were also expressed. These findings are similar to the writings of Butler-Williams et al. (2010) that state that the close proximity of HCAs to patients has led them to be described as ‘insightful observers’ and that they are well-positioned to identify early warning signs of deterioration.

Simultaneously, the growing distance of PNs from patients and their decreasing involvement in direct patient care were revealed. This aligns with the findings of Furåker (2008) and Keeney et al. (2005) that nurses’ work is shifting towards more indirect care activities, such as coordination, supervision and administrative work. Keeney et al. (2005) stated that effective employment of HCAs requires that their work is directed and supervised by registered nurses. Butler-Williams et al. (2010) further stated that the responsibility of the registered nurse to provide supervision at the bedside should be assured. Yet, both nurses and UMs acknowledged that PCWs most often worked on their own without supervision. This corresponds with the findings of Snell in McKenna et al. (2004), indicating that 53% of HCAs reported that little or none of their work was supervised. Poor supervision of HCAs’ work exposes both nurses and employers to legal and ethical risks.

As part of the tasks delegated to PCWs by PNs, most PCWs reported that they knew what to do and followed routines through which the hygiene and comfort needs of patients were addressed. All participants further reported that PCWs were included in handover patient discussion meetings. This finding does not align to the notation of Spilsbury and Meyer (2004) that the roles of HCAs are sometimes limited by nurses by not involving them in discussions concerning patient care.

The findings of this study illustrated that nurses acknowledged, valued and relied on the ability of PCWs to report abnormalities and that PCWs documented their observations and activities on patient documents. According to Hancock and Campbell (2006), the inclusion of PCWs in documentation and patient care meetings conveys respect to them. However, Spilsbury and Meyer (2004) found that the passing on of HCAs’ observations was not actively sought and valued or acknowledged by nurses.

Patient care workers are legally not acknowledged as nurses, as they are not registered according to the *South African Nursing Act*, No. 33 of 2005. Yet, most participants expressed that they regarded PCWs as nurses and that they were actually rendering nursing care. Nurses, therefore, do not seem to understand the legal regulation of their practice or the implications thereof for nursing practice.

Most participants were of the opinion that there was little difference between the ENA’s and the PCW’s roles. Most of the direct care activities mentioned by participants seem similar to the definition of elementary nursing, which falls within the domain of the ENA. The ENAs one-year training programme and practice as a nurse is regulated (South African Nursing Council 1993). The activities of PCWs thus overlap with those of ENAs although their training courses are shorter and not standardised. The role boundary between nurses and PCWs, therefore, does not appear distinctive.

Several authors stressed financial pressure as a reason for the employment of HCAs (Armstrong et al. 2013; Hunt 2010; Keeney et al. 2005). However, only three participants offered financial restrictions as a reason for the employment of PCWs. It was mentioned that there was pressure on staff to stay within the budget and that PCWs were seen as ‘cheap labour’ or ‘cheaper’ to hire than ENAs. The World Medical Association (WMA) general assembly for task shifting from the medical profession poses a warning that task shifting should not be viewed solely as a cost-saving measure, because the economic benefits of task shifting have not been substantiated and that such cost-driven methods are unlikely to produce quality results that will serve the best interests of patients. The resolution further states that the aspiration should be to train more skilled professionals, rather than shifting tasks to less skilled workers (WMA Assembly 2009).

Furthermore, the literature is quite pertinent that the HCA’s function has grown in importance because of the continuing nursing recruitment and retention crisis (Keeney et al. 2005). However, nursing shortage as such was never mentioned by participants. Yet, participants often explained that PCWs did those things that the nurses could not get to. Specific tasks, related to direct care, were shifted from nurses to PCWs to ease the workload of nurses. The World Health Organization (WHO) cautions that task shifting should be implemented within systems that contain adequate control and evaluation checks to protect both health workers and patients (WHO 2007).

Most participants mentioned concerns that PCWs did more than they were allowed to do, similar to the findings by Spilsbury et al. (2009) which support that HCAs’ responsibility for patient care is increasing. UMs were particularly concerned that, although PCWs might be able to do the tasks, they lacked the knowledge and insights to understand the possible consequences of some of their actions. Kalisch (2009) similarly described the concern of nurses about the training and performance of HCAs, and that nurses are hesitant to delegate to HCAs as they do not trust their competency levels. Regulation or credentialing is advised to enable and to regulate task shifting, as well as to ensure that the new type of health workers are appropriately qualified for the tasks that they will undertake (WHO 2007).

Informed decisions regarding the deployment and further management of PCWs are essential for optimal staff utilisation and safe patient care. This study provides a description of their role in private hospitals to support informed decision-making.

## Limitations of the study

The study was conducted in medical and surgical wards of private hospitals and excluded other units, such as critical care units and paediatric wards. Suggestions for further research include the experience patients have regarding the role of PCWs and the effect of PCWs’ employment on the quality of patient care.

## Implications for nursing management and recommendations

The activities of PCWs seem similar to elementary care, which is the domain of the ENA and, therefore, overlap with the ENA’s scope of practice. PCWs are not regulated and, therefore, their training programmes and activities in hospitals vary. They are also not legally entitled to practise nursing in South Africa. Their competency to care for patients in the hospital setting and their insight to observe and report on the patient’s condition are questioned, although nurses relied on PCWs to report abnormalities and expressed that PCWs were good observers.

According to Spilsbury et al. (2009) regulation of PCWs would help to standardise their role, while also providing recognition, confidence and career progression to PCWs (WHO 2007). Stellenberg and Dorse (2014) further stress the importance of regulation to align formal training to legislation and to protect the public. The importance of regulation to ensure patient safety is also described by Hand (2012). An appropriate legislative framework will further protect nurses, as nurses who delegate functions to PCWs will remain accountable should adverse events happen. The work of PCWs is similar to ENAs; therefore, the employment of ENAs is recommended as this is a regulated category.

Furthermore, budget restrictions were mentioned as a reason for the employment of PCWs. Task shifting should not be viewed solely as a cost-saving exercise as the economic benefits of task shifting have not been substantiated and the benefits to patients are unlikely (WMA Assembly 2009).

Nurses seemed to be unclear on the legislation governing the nursing profession and struggled to explain the practical implications of relevant legislation accurately. Nurses should regularly be updated on relevant legislation, while new regulations after publication should be included in nurses’ continuous development programmes.

## Conclusion

Patient care workers have been a part of patient care teams in hospitals for several years and their role has evolved over the years. It is, therefore, essential to critically evaluate their role as their participation in nursing care cannot be ignored.
